# Simultaneous Evaluation of Shear Wave Elastography and C-Peptide Index for Predicting Need of Insulin Therapy in Type 2 Diabetes: A Pilot Study

**DOI:** 10.3390/jpm15070277

**Published:** 2025-07-01

**Authors:** Moeno Sugita-Hamada, Takeshi Yokoo, Nao Nakajima, Yoshifumi Takahashi, Akihiko Osaki, Masaki Maruyama, Masaaki Takamura, Nobuo Waguri, Osamu Isokawa, Shuji Terai

**Affiliations:** 1 Division of Gastroenterology and Hepatology, Graduate School of Medical and Dental Sciences, Niigata University, Niigata 951-8510, Japan; 2 Department of Preemptive Medicine for Digestive Diseases and Healthy Active Life, Graduate School of Medical and Dental Sciences, Niigata University, Niigata 951-8510, Japan; 3 Department of Gastroenterology, Kashiwazaki General Hospital and Medical Center, Kashiwazaki 945-8535, Japan; n-nakaji@med.niigata-u.ac.jp (N.N.); marumasa@kashiwazaki-ghmc.jp (M.M.);; 4 Department of Gastroenterology and Hepatology, Niigata City General Hospital, Niigata 950-1197, Japan

**Keywords:** shear wave elastography, shear wave dispersion, type 2 diabetes mellitus, C-peptide index, pancreatic stiffness

## Abstract

**Background/Objectives**: Recently, shear wave elastography (SWE) and dispersion (SWD) targeting the pancreas have been attempted as noninvasive procedures to evaluate personalized conditions. This study aimed to analyze the feasibility of utilizing them for evaluating the individual need of introducing insulin therapy, combined with the C-peptide index (CPI), in patients with type 2 diabetes mellitus (T2DM). **Methods**: This study involved 51 patients with T2DM aged ≥20 years old and 20 control subjects without impaired glucose tolerance (CTRL). T2DM were divided into non-insulin-treated (non-INS) and insulin-treated (INS) groups. Their background data, shear wave speed (SWS), and dispersion slope (DS) of the pancreas were obtained on the same day. **Results**: Pancreatic SWS was higher in T2DM than in CTRL (*p* < 0.0001), with an AUC of 0.840, sensitivity of 89.1%, and specificity of 70.6%, using a Youden index cutoff of 1.31 m/s. INS and non-INS were discriminated with the cutoff value of 1.70 m/s (*p* = 0.031, AUC 0.736, sensitivity 55.6% and specificity 89.2%). Pancreatic DS of INS and non-INS was 13.52 and 12.16 (m/s)/kHz, respectively (*p* = 0.046). Using 12.38 (m/s)/kHz as the cutoff, AUC was 0.718, with sensitivity of 88.9%, specificity of 56.8% and negative predictive value of 95.5%. CPI had AUC of 0.724, sensitivity of 66.7% and specificity of 83.3% with the cutoff of 0.63. With combination of SWS and CPI, all patients with SWS < 1.70 m/s and CPI > 0.476 belonged to non-INS. **Conclusions**: Simultaneous non-invasive SWE and CPI evaluation showed the feasibility for estimating personalized insulin initiation needs in T2DM, integrating biophysical and hormonal perspectives. Further investigation with a larger, multi-center study population is warranted to enhance the level of evidence.

## 1. Introduction

Patients with type 2 diabetes mellitus (T2DM) have a decreased quality of life (QOL) due to micro- and macrovascular complications such as myocardial infraction, renal and vision impairment, gangrene, and necessity for amputation. To prevent the loss of QOL, algorithms for pharmacotherapy were proposed by the Japan Diabetes Society in 2022 [1] and the American Diabetes Association in 2024 [2]. In those algorithms, marked hyperglycemia at diagnosis (HbA1c ≥ 10% or Fasting Plasma Glucose ≥ 300 mg/dL) or symptomatic hyperglycemia (polyuria, polydipsia, weight loss) are referred to as barometers of insulin initiation. Although hormonal indices such as C-peptide index (CPI) [3,4,5,6] have been used to assess the need for introduction of insulin therapy in patients with T2DM, histological evaluation has been deemed impractical due to its highly invasive nature.

Non-invasive examinations using ultrasound systems to obtain pathological information have become increasingly prevalent across various institutions. These examinations can be performed not only by medical doctors but also by ultrasound technicians. In particular, shear wave elastography (SWE), quantified as shear wave speed (SWS), has been extensively studied, and its efficacy is being evaluated in numerous organs. While the liver is a well-established application [7], non-hepatic applications have also been reported in a wide range of organs, including the pancreas, breast, prostate, thyroid, gastrointestinal tract, spleen, kidney, lymph nodes, skeletal muscle, testis, and even blood vessels [8]. Similarly, shear wave dispersion (SWD) is gaining attention as a novel non-invasive method, assessed by its dispersion slope (DS). Unlike SWS, which primarily reflects tissue elasticity derived from fibrosis, SWD is considered a potential indicator of tissue viscosity. Key factors influencing tissue viscosity is reported to include inflammation and necrosis within the tissue [9,10].

SWE and SWD of the pancreatic parenchyma have been shown to be a noninvasive and reproducible histological evaluation [8,11,12,13]. In pancreatic stiffness measurements in T2DM, high SWS was reported to be a risk for microangiopathy [14,15], and a study [16] of autoimmune pancreatitis in a pancreas showed higher SWS and DS than in a healthy pancreas, both of which exhibited declines following the administration of prednisolone. These parameters have great potential for evaluating the pathological condition of pancreatic diseases.

Given the long-term nature of T2DM, the non-invasive nature of SWE and SWD offers a significant advantage for the longitudinal evaluation of pancreatic conditions. Regular assessment using these techniques may guide treatment decisions or adjustments, and could contribute to the evaluation of comorbidity risk. Appropriate longitudinal monitoring of the pathophysiological state has the potential to improve both QOL and prognosis. However, the utility of these methods for this purpose has not been sufficiently investigated to date.

In this study, we aimed to analyze the feasibility of utilizing SWE and SWD to evaluate the individual need for initiating insulin therapy in patients with type 2 diabetes mellitus (T2DM). Furthermore, as a reasonable and promising strategy that focuses on both pancreatic histological findings and insulin secretory reserve, we also analyzed the feasibility of simultaneously evaluating SWE and the C-peptide index (CPI).

## 2. Methods and Datasets

This was a retrospective observational study. Subjects were 71 Japanese individuals whose pancreatic SWS was measured between December 2018 and February 2021 at Kashiwazaki General Hospital and Medical Center or Niigata City General Hospital. The dataset was obtained in a preceding prospective observational study evaluating the relationship between insulin secretion and ultrasound findings such as SWS, DS and attenuation imaging. In the current study, this dataset was secondarily used and analyzed from a perspective of data combination in more details. The secondary use of information from the preceding study was disclosed, and participants were requested to contact us if they wished to refuse. There were no requests for refusal of participation.

Fifty-one people had already been diagnosed as T2DM. The other 20 people did not have impaired glucose tolerance. The diagnosis of T2DM was made according to a consensus statement from the Japan Diabetes Society published in 2022 [1]. In thirteen out of 51 T2DM patients, insulin therapy had been started based on the consensus statement, in which HbA1c, fasting plasma glucose and symptomatic hyperglycemia were recommended as indicators for insulin initiation.

To ensure the reproducibility of SWE, the top 5% (3 cases) exhibiting the highest robust coefficients of variation (%rCV) in pancreatic stiffness measurements were excluded. Furthermore, to mitigate the potential influence of pancreatic calcification and congestion on SWS and DS measurements, which could lead to an overestimation of pancreatic SWS, 5 cases with conditions such as chronic pancreatitis, alcohol use disorder, liver cirrhosis, and congestive heart failure were excluded. Following the exclusion of these 8 cases, the data from 17 control subjects without impaired glucose tolerance (CTRL) and 46 patients with type 2 diabetes mellitus (T2DM) were analyzed.

**Figure 1:**
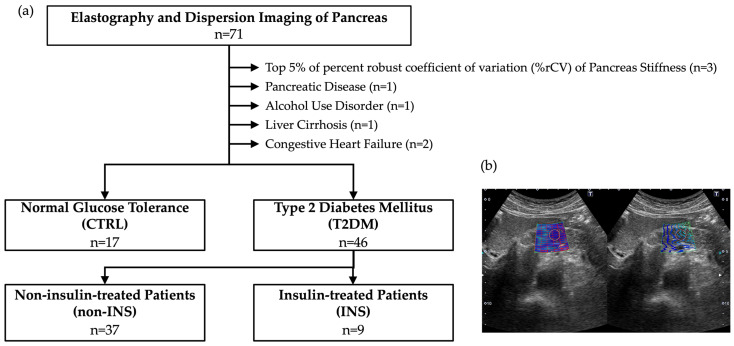
Enrollment of subjects and measurement of shear wave speed and dispersion slope on the pancreas. (**a**) Enrollment of subjects. (**b**) Measurement of shear wave speed (SWS) and dispersion slope (DS). Measurements were performed at the pancreatic head or body in the spine position after 12 h of fasting. The distribution of SWS was shown as a color map in the left panel. A region of interest was 10 mm in diameter (Circles in the left and right panels). SWS and DS were measured in a total of five locations with appropriate propagation maps (The right panel), and median value and the percent robust coefficient of variation (%rCV) were calculated, respectively.

The T2DM patient population was further divided into 9 patients receiving regular insulin therapy (INS), and 37 patients not receiving regular insulin therapy (non-INS) at the point of abdominal ultrasonography (Figure 1a).

Age, sex, height, and weight were recorded for all subjects. In addition, SWS and DS, serum amylase, fasting glucose, HbA1c, total cholesterol, triglycerides, eGFR, platelet count, AST, ALT, and fasting C-peptide were recorded in T2DM on the same day as a measurement of SWS and DS. BMI, CPI [3,4,5,6], FIB-4 index [17], and APRI [18] were calculated based on these data. CPI was calculated by “fasting serum C-peptide [ng/mL] ÷ fasting serum glucose [mg/dL] × 100”. FIB-4 index was calculated by “age [years] × AST [U/L]/(platelets [10^9^/L] × (ALT [U/L])^1/2^)”. APRI is AST to Platelet Ratio Index, which was calculated by “AST [U/L]/(platelets [10^9^/L]”. Furthermore, usage of oral antidiabetic drugs was investigated. All data were obtained at Kashiwazaki General Hospital and Medical Center or Niigata City General Hospital and analyzed at Niigata University.

SWE and SWD of the pancreas were performed by five hepatologists who were trained in advance. The ultrasound device used was a Canon Medical Systems Aplio i800 ultrasound system equipped with a 4.0 Hz convex probe (PVI-4758BX). Measurements were performed with patients in the supine position after fasting for at least 12 h. As normal pancreatic tissue is known to exhibit uniform stiffness regardless of the site examined [8], in this study, measurements were performed using a 10 mm circular ROI at 5 arbitrary points on the pancreatic head and body (Figure 1b). During a breath-hold in the spontaneous expiratory position, SWS and DS were simultaneously acquired by pressing a measurement button. The reliability of each individual measurement was assessed using Aplio’s unique propagation map, which was generated concurrently with the SWS and DS measurements. This propagation map was displayed at the same time of the SWS and DS measurements and facilitated the evaluation of elastogram quality. Subsequently, the median values of SWS, DS, and %rCV were calculated from five reliable measurements. To investigate the ability of SWS, DS, and CPI to discriminate between INS and non-INS cases, Receiver Operating Characteristic (ROC) curves were generated, and sensitivity and specificity were determined based on the Youden index.

All statistical analyses were performed using EZR software (version 1.60) [19]. Measured data were expressed as median values with the first and third quartiles, and comparisons of continuous variables between two groups were performed with non-parametric tests (Mann–Whitney U test). Statistical significance was defined as *p* < 0.05. AUC comparisons were performed with the Bootstrap test for two correlated ROC curves.

## 3. Results

### 3.1. Comparison of SWS Between T2DM and CTRL

**Figure 2:**
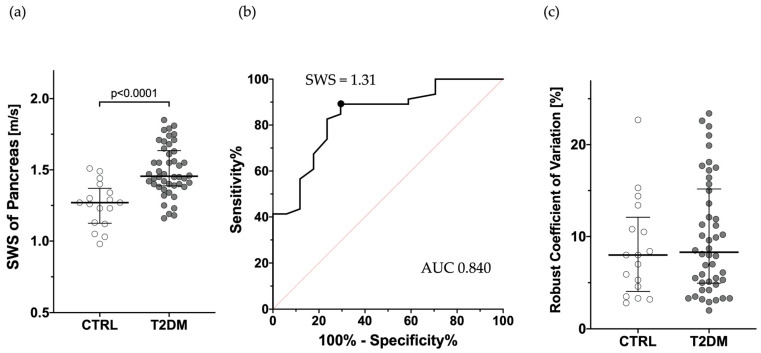
Comparison of shear wave elastography between type 2 diabetes mellitus (T2DM) and control (CTRL). (**a**) Comparison of shear wave speed (SWS) of the pancreas between T2DM and CTRL. (**b**) Discrimination power between T2DM and CTRL. (**c**) Percent robust coefficient of variation (%rCV) for T2DM and CTRL. Gray circles, T2DM; white circles, CTRL.

The median age with interquartile range (IQR) of T2DM and CTRL were 68 (60–71) and 61 (51–68) years old, respectively, with BMIs of 23.4 kg/m^2^ (21.1–26.6) and 22.7 kg/m^2^ (21.1–23.8). No significant difference was found between the two groups. No patient had severe obesity which resulted in unclear image of the pancreas. The male-to-female ratio was significantly higher in T2DM (67.4% vs. 29.4%, *p* = 0.010). SWS was significantly higher in the T2DM group (1.46 m/s with IQR of 1.39–1.62) compared to the control (CTRL) group (1.27 m/s with IQR of 1.13–1.34) (*p* < 0.0001, Figure 2a). AUC was 0.840, with a 95% confidence interval of 0.731–0.949 (Figure 2b), and when the cutoff value was set at 1.31 m/s based on the Youden index, the sensitivity was 70.6% and specificity was 89.1%. The median robust coefficient of variation (%rCV) calculated from the measurements of the five sites was 8.30 (5.06–14.69) and 7.96 (4.60–10.85), respectively, with no significant difference (Figure 2c). These results suggest that SWS can be reproducibly measured in T2DM as well as in CTRL, and that SWE can discriminate T2DM from CTRL with high accuracy.

### 3.2. Comparison of SWS, DS, and CPI in Discriminating INS from Non-INS Patients

**Figure 3:**
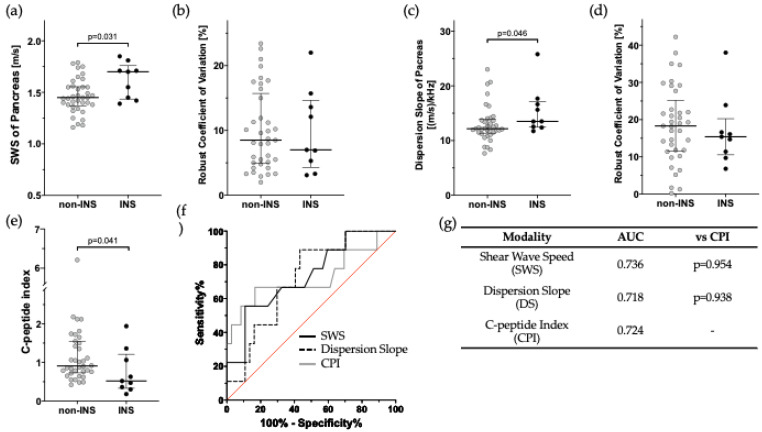
Comparison of shear wave speed (SWS) and dispersion slope (DS) between insulin-treated patients (INS) and non-insulin-treated patients (non-INS). (**a**,**b**) SWS and percent robust coefficient of variation (%rCV) for INS and non-INS. (**c**,**d**) DS and %rCV of INS and non-INS. (**e**) C-peptide Index (CPI) of INS and non-INS. (**f**,**g**) Comparison of discrimination power among SWS, DS, and CPI. Black circles, INS: light gray circles, non-INS: black line, SWS: black dotted line, DS: gray line, CPI.

Table 1 shows the backgrounds of the INS and non-INS patients. No significant differences were observed between the two groups except for the male/female ratio and HbA1c. SWE results showed that the median SWS for INS and non-INS was 1.70 m/s and 1.45 m/s, respectively (*p* = 0.031), as shown in Figure 3a. The %rCV of SWS did not differ between the two groups (Figure 3b). Similarly, SWD results showed that the median DS was 13.51 (m/s)/kHz and 12.16 (m/s)/kHz (*p* = 0.046, Figure 3c). The %rCV of DS did not differ between the two groups (Figure 3d). On the other hand, CPI was INS 0.52 and non-INS 0.90 (*p* = 0.041, Figure 3e). AUC for SWS and DS were 0.736 and 0.718, which did not statistically differ compared to 0.724 for CPI (Figure 3f,g). These results suggest that SWS, DS, and CPI all have the ability to discriminate INS from non-INS, but it was not so sufficient to utilize them in a clinical diagnosis.

Regarding oral antidiabetic drugs, glucagon-like peptide-1 receptor agonists (GLP-1 RAs), a class of incretin-based therapies, have been reported to be associated with structural changes in the pancreas [20]. Although GLP-1 agonist use had the potential to influence SWS and DS measurements, there was no significant difference in their usage between the INS and non-INS groups in this study (0% vs. 2.7%). The percentage of dipeptidyl peptidase-4 (DPP-4) inhibitor use, another incretin-related drug class, was 77.8% in the INS group and 75.7% in the non-INS group.

### 3.3. Usefulness of Combined Use of SWS and CPI

**Figure 4:**
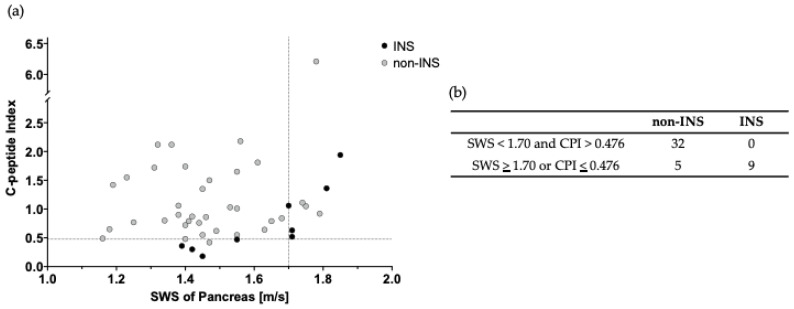
Distribution of insulin-treated patients (INS) from non-insulin-treated patients (non-INS) and cut-off values to discriminate INS from non-INS. (**a**) Plotting INS and non-INS. The X– and Y–axes show shear wave speed (SWS) and C-peptide index (CPI), respectively. (**b**) Distribution numbers of INS and non-INS. Black circles, INS; light gray circles, non-INS; dotted lines, cut-off values.

As shown in Table 2, the specificity of SWS and CPI for discriminating INS from non-INS was high at 89.2% and 83.3%, respectively, using the cutoff values calculated from the Youden index. However, the sensitivity was low at 55.6% and 66.7%, respectively. In contrast, while the sensitivity of DS was high at 88.9%, its specificity was only 56.8%. Given that these results were not sufficient for clinical application, we attempted to discriminate INS from non-INS by using SWS and CPI in combination. Using the scatterplot in Figure 4a,b as a reference, the sensitivity, specificity, and accuracy of SWS and CPI were 100%, 86.5%, and 89.1%, respectively, with cutoff values of 1.69 m/s for SWS and 0.48 for CPI, indicating high discrimination performance.

## 4. Discussion

In this study, we attempted to evaluate the insulin secretory capacity of T2DM using SWE and SWD. Although SWE and SWD alone had the same diagnostic performance as the conventionally used CPI [3,4,5,6], they did not have sufficient diagnostic accuracy. Therefore, we combined SWS and CPI as a hormonal and pathological approach, and obtained promising results with a sensitivity of 100%, specificity of 86.5%, and accuracy of 89.1%. Measurements of SWS and CPI can aid in the decision-making and modification of individual treatment plans. In patients not currently receiving insulin therapy, an SWS value below 1.70 m/s combined with a CPI value above 0.476 indicates a low risk of future insulin initiation with high sensitivity. Conversely, an SWS value above 1.70 m/s or a CPI value below 0.476 suggests the need for modification of the individual treatment plan to potentially reduce the risk of insulin initiation. We presume that our proposed cutoff values are optimal for identifying patients with a low likelihood of requiring insulin initiation.

Abdominal ultrasound is not a recognized or standard screening tool for diagnosing T2DM itself. However, it is very useful tool to assess comorbidities associated with T2DM, especially non-alcoholic fatty liver disease (NAFLD) or metabolic dysfunction-associated steatotic liver disease (MASLD). The higher comorbidity and cancer-related mortality [21] in this population support its role in screening for associated complications, not for diabetes itself. SWE and SWD can be measured as part of series of routine observation.

From a literary point of view, the mechanism by which SWE reflected the need of insulin initiation can be explained by amyloid deposition. In T2DM, amyloid deposition has long been known in the pancreas [22]. This amyloid is known as islet amyloid polypeptide (IAPP) and plays a physiological role in glucose homeostasis by suppressing glucagon release, controlling gastric emptying, and regulating satiety [23]. The amount of IAPP deposition has been negatively correlated to β-cell volume [24,25]. Indeed, Changes in organ stiffness due to amyloid deposition in organs have been reported. In 2010, usefulness of vibration-controlled transient elastography (VCTE) was suggested in a patient with histologically diagnosed hepatic amyloidosis by transjugular liver biopsy (TJLB) [26]. No accompanying fibrosis was confirmed in the liver of the patient, suggesting that the amyloid deposition itself may have increased liver stiffness. In a report of familial Mediterranean fever, two dimensional SWE in both the liver and pancreas could reflect concomitant deposition of amyloid [27]. Similarly, MR elastography (MRE) results in extreme liver stiffness in biopsy-proven liver amyloidosis [28]. It was reported that improvement of liver stiffness in VCTE resulted from removal of amyloid by treatment with an antibody to serum amyloid P component [29]. This strongly suggests an association between amyloid deposition and organ stiffness. In addition, usefulness of liver stiffness measurement was indicated in 41 and 10 patients with hepatic amyloidosis utilizing VCTE and MRE [30,31]. Therefore, evaluating amyloid deposition in the pancreas through noninvasive stiffness measurement appears to be reasonable.

Pancreatic histology has not been applied to T2DM in routine clinical practice due to the invasive nature in living patients, therefore, autopsy specimens have been used for pathological analysis. While examining autopsy specimens from deceased patients holds academic importance, it offers no direct benefit to the deceased individual. In contrast, SWE and SWD are non-invasive and can be repeatedly performed on living patients for regular monitoring. Given the typically long duration of T2DM, longitudinal evaluation of the pancreatic condition is necessary and significant for guiding lifestyle interventions and determining therapeutic strategies. Therefore, the non-invasive and repeatable nature of SWE and SWD are considered a substantial advantage, even though clinicians cannot directly observe and evaluate pancreatic tissue using these techniques.

Beyond their non-invasive nature, SWE and SWD present a significant advantage by offering a means to quantify the biophysical state of pancreatic tissue. This capability stands in stark contrast to the information derived from traditional pathological analysis, wherein microscopic examination of tissue biopsies typically yields findings that are qualitative in description or at best, semi-quantitative in their assessment. The continuous and objective data streams generated through SWE and SWD possess the inherent potential to detect and characterize even subtle alterations within the pancreatic parenchyma, changes that might be below the threshold of detection using conventional histopathological techniques. This intriguing hypothesis, suggesting a superior sensitivity to early or nuanced tissue modifications, warrants further, comprehensive investigation in larger cohorts.

Moreover, SWE and SWD boast a considerable advantage in their inherent objectivity, particularly when juxtaposed with the inherent subjectivity associated with pathological interpretation. The process by which pathologists analyze and interpret tissue samples necessitates a degree of subjective judgment, a process deeply rooted in their individual accumulated experience and training. Consequently, this reliance on individual interpretation makes the avoidance of inter-rater variability, or discrepancies in assessment between different pathologists, a persistent challenge within the field. Similarly, the potential for intra-rater variability, where a single pathologist might render slightly different interpretations of the same sample at different times, also constitutes a recognized limitation. While ultrasound-based examinations, including SWE and SWD, are not entirely immune to variability arising from technical factors or operator dependence, the inherent quantitative nature of their output allows for mitigation strategies. Specifically, the acquisition of multiple measurements followed by the calculation of a median value serves as a robust method to minimize the impact of random variations and enhance the overall reliability and consistency of the assessment.

This study holds significance as it suggests that the simultaneous measurement of SWS and CPI has the potential to evaluate the need for insulin initiation with higher sensitivity compared to the individual use of parameters such as SWE, SWD, and CPI. Given the pancreas’s challenging nature for examination, a multi-faceted approach is essential for diagnosing its conditions. Our proposed approach integrates hormonal and biophysical assessments, enabling clinicians to evaluate the pancreas both functionally and morphologically.

Furthermore, this non-invasive method has potential applications not only in insulin therapy initiation but also across all phases of T2DM progression and amelioration, including impaired glucose tolerance. In routine clinical practice, it is crucial to prevent the onset of T2DM as well as to manage the chronic and often intractable nature of established T2DM. As a future direction, it will be necessary to investigate whether the simultaneous measurement of SWS and CPI can identify impaired glucose tolerance prior to the development of T2DM. This preemptive medicine perspective may offer benefits in terms of healthcare economics and the prolongation of healthy life expectancy.

This study has several limitations, including its non-blind and retrospective design, the small sample size, and the absence of external validation. Generally, specificity and sensitivity can be artificially high and low, respectively, when the number of true positive samples is small. From this perspective, further investigation with a larger, multi-center study population is necessary to enhance the level of evidence. A Longitudinal study may be significant to determine whether this method can effectively assess the progression and amelioration of T2DM over time. Ideally, future research should explore the histological correlates of increased SWS and DS, however, verification is challenging due to the invasive nature of tissue sampling. The influence of oral antidiabetic drugs, particularly GLP-1 RAs, warrants in-depth investigation, given their reported association with structural changes in the pancreas [20]. Similarly, the impact of DPP-4 inhibitors, another class of incretin-related drugs, should also be evaluated. While the usage of GLP-1 RAs and DPP-4 inhibitors was equivalent between the INS and non-INS groups in our cohort, suggesting the reliability of the comparison between these groups, the potential influence of incretin-related drugs on SWS in T2DM patients remains unclear.

## 5. Conclusions

In conclusion, the simultaneous evaluation of non-invasive SWE and CPI suggests the feasibility for estimating the personalized need for insulin initiation in patients with T2DM, integrating both biophysical and hormonal perspectives. Further investigation in a large, multi-center population is warranted to enhance the level of evidence.

## Figures and Tables

**Table 1:** Background characteristics of type 2 diabetes mellitus (T2DM) patients.

	T2DM (*n* = 46)			
		Non-INS (*n* = 37)	INS (*n* = 9)	*p* Value
Age [y.o.]	68 (60–71)	68 (60–71)	65 (59–71)	not significant (n.s.)
M/F	31/15	29/8	2/7	0.003
BMI [kg/m^2^]	23.4 (21.1–26.6)	23.5 (21.6–26.7)	22.2 (19.7–24.0)	n.s.
AMY [U/L]	78 (70–97)	83 (70–106)	70 (56–77)	n.s.
HbA1c [%]	7.5 (7.0–8.1)	7.3 (6.9–8.0)	8.1 (7.9–8.6)	0.003
TC [mg/dL]	200 (183–222)	197 (183–215)	207 (199–226)	n.s.
TG [mg/dL]	114 (73–157)	113 (78–149)	134 (55–274)	n.s.
eGFR [mL/min/1.73 m^2^]	69.70 (57.68–80.49)	70.25 (58.55–80.34)	65.72 (56.15–82.28)	n.s.
PLT [×10^4^/μL]	24.8 (21.3–27.5)	24.7 (20.3–26.5)	28.2 (22.1–28.9)	n.s.
AST [U/L]	19 (17–22)	19 (17–22)	18 (15–20)	n.s.
ALT [U/L]	17 (14–22)	17 (14–21)	17 (14–22)	n.s.
FIB-4 index	1.20 (0.98–1.59)	1.25 (1.02–1.60)	1.02 (0.88–1.31)	n.s.
APRI	0.27 (0.21–0.40)	0.28 (0.22–0.43)	0.24 (0.18–0.31)	n.s.
SWS of the Liver	1.40 (1.32–1.53)	1.44 (1.33–1.54)	1.35 (1.29–1.45)	n.s.

**Table 2:** The power of the combination of shear wave speed (SWS), dispersion slope (DS), and C-peptide index (CPI) for discriminating INS from non-INS.

Modality	Cut-off Value	Sensitivity [%]	Specificity [%]	Positive Predictive Value [%]	Negative Predictive Value [%]	Accuracy [%]
Shear wave speed (SWS)	1.70	55.6	89.2	55.6	89.2	82.6
Dispersion slope (DS)	12.38	88.9	56.8	33.3	95.5	63.0
C-peptide index (CPI)	0.63	66.7	83.3	50.0	91.2	80.4
SWS and CPI	1.69 & 0.48	100	86.5	64.3	100	89.1

## Data Availability

The data presented in this study are available on request from the corresponding author. The data are not publicly available due to ethical restrictions.

## Abbreviations

The following abbreviations are used in this manuscript:

T2DM	type 2 diabetes mellitus
CPI	C-peptide index
SWE	shear wave elastography
SWD	shear wave dispersion
SWS	shear wave speed
DS	dispersion slope
%rCV	robust coefficients of variation
CTRL	control subjects without impaired glucose tolerance
INS	patients receiving regular insulin
non-INS	patients not receiving regular insulin
